# The Innate Immune Response to Infection Induces Erythropoietin-Dependent Replenishment of the Dendritic Cell Compartment

**DOI:** 10.3389/fimmu.2020.01627

**Published:** 2020-07-31

**Authors:** Henrik Einwächter, Alexander Heiseke, Andreas Schlitzer, Georg Gasteiger, Heiko Adler, David Voehringer, Markus G. Manz, Zsolt Ruzsics, Lars Dölken, Ulrich H. Koszinowski, Tim Sparwasser, Wolfgang Reindl, Stefan Jordan

**Affiliations:** ^1^II. Medizinische Klinik, Klinikum Rechts der Isar, Technische Universität München, Munich, Germany; ^2^LIMES-Institute Bonn, Bonn, Germany; ^3^Institute of Systems Immunology, Julius-Maximilians-University Würzburg, Würzburg, Germany; ^4^Comprehensive Pneumology Center, Research Unit Lung Repair and Regeneration, Helmholtz Zentrum München - German Research Center for Environmental Health (GmbH), Munich, Germany; ^5^German Center of Lung Research (DZL), Giessen, Germany; ^6^Department of Infection Biology, Universitätsklinikum Erlangen and Friedrich-Alexander-Universität Nürnberg, Erlangen, Germany; ^7^Division of Hematology, Department of Internal Medicine, University Hospital Zurich, Zurich, Switzerland; ^8^Institute of Virology, University Medical Center, Medical Faculty, University of Freiburg, Freiburg, Germany; ^9^Institute for Virology and Immunobiology, Julius-Maximilians-University Würzburg, Würzburg, Germany; ^10^Max von Pettenkofer-Institute, Ludwig-Maximilians-Universität München, Munich, Germany; ^11^Institute of Medical Microbiology and Hygiene, University Medicine Mainz, Johannes-Gutenberg-University Mainz, Mainz, Germany; ^12^II. Medizinische Klinik, Medizinische Fakultät Mannheim, Universität Heidelberg, Mannheim, Germany; ^13^Charité – Universitätsmedizin Berlin, Corporate Member of Freie Universität Berlin, Humboldt-Universität zu Berlin, and Berlin Institute of Health, Institute of Microbiology, Infectious Diseases and Immunology, Berlin, Germany

**Keywords:** infection, inflammation, erythropoietin, dendritic cell, extramedullary hematopoiesis, TER119, toll-like receptor

## Abstract

Dendritic cells (DC) play a key role in the adaptive immune response due to their ability to present antigens and stimulate naïve T cells. Many bacteria and viruses can efficiently target DC, resulting in impairment of their immunostimulatory function or elimination. Hence, the DC compartment requires replenishment following infection to ensure continued operational readiness of the adaptive immune system. Here, we investigated the molecular and cellular mechanisms of inflammation-induced DC generation. We found that infection with viral and bacterial pathogens as well as Toll-like receptor 9 (TLR9) ligation with CpG-oligodeoxynucleotide (CpG-ODN) expanded an erythropoietin (EPO)-dependent TER119^+^CD11a^+^ cell population in the spleen that had the capacity to differentiate into TER119^+^CD11c^high^ and TER119^−^CD11c^high^ cells both *in vitro* and *in vivo*. TER119^+^CD11c^high^ cells contributed to the conventional DC pool in the spleen and specifically increased in lymph nodes draining the site of local inflammation. Our results reveal a so far undescribed inflammatory EPO-dependent pathway of DC differentiation and establish a mechanistic link between innate immune recognition of potential immunosuppressive pathogens and the maintenance of the DC pool during and after infection.

## Introduction

Dendritic cells (DC) are professional antigen-presenting cells crucial for coordinating and regulating immune responses. The ability of DC to prime naïve T cells depends on the antigen-presenting function of DC, but also on their ability to provide stimulating cytokines. The origin and the development of DC has been intensively studied (1). Based on their ontogeny, the DC family can be subdivided into four cell types: Langerhans cells, plasmacytoid DC (pDC), conventional DC (cDC) and monocyte-derived DC (2). Langerhans cells are seeded in the skin during embryonic development or derive from monocytes during inflammation (3, 4). cDC and pDC both originate from a pluripotent hematopoietic stem cell in the bone marrow (5). pDC fully develop in the bone marrow and only mature in the periphery (6). cDC differentiate via bone marrow-resident common myeloid progenitors (CMP), macrophage DC progenitors (MDP), and common DC progenitors (CDP) into pre-DC that leave the bone marrow to the blood circulation and seed peripheral tissues (5–8). In the periphery, cDC fully differentiate into cDC subpopulations cDC1 and cDC2 depending on the transcription factors BATF3 and IRF4 (9), respectively, and acquire subset-specific functions. In mouse spleen, cDC subpopulations can be distinguished by a CD4^−^CD8^+^CD11b^−^ (cDC1) and CD4^+^CD8^−^CD11b^+^ (cDC2) surface phenotype (2). Monocyte-derived DC develop under inflammatory conditions from an early myeloid progenitor cell (8). It is important to note that even during later stages of lineage commitment DC development retains a certain plasticity, as for example pDC precursors can re-differentiate into cDC in the periphery (10).

Many bacteria (e.g., *Salmonella enterica, Yersinia enterolytica, Helicobacter pylori*) (11) and viruses (e.g., Human Immunodeficiency Virus (HIV), herpesviruses, paramyxoviruses) (12–15) interfere with the initiation of an adaptive immune response by targeting or even eliminating DC. Also, the number of splenic DC is critically reduced in severe polymicrobial infections (16). Hence, it is desirable to replenish the DC pool as early as possible in the course of an infection in order to keep the adaptive immune system effective. The recognition of viral and bacterial pathogens by pattern recognition receptors of the innate immune system triggers a robust inflammatory response that increases the numbers of hematopoietic stem and progenitor cells, resulting in expansion of myeloid lineages (17). Interestingly, inflammatory activation of the hematopoietic system is not limited to the bone marrow, but certain infections can induce the egress of early progenitor cells from the bone marrow to the spleen or expand spleen-resident progenitor cells to allow for extramedullary hematopoiesis (18–20). Extramedullary hematopoiesis critically depends on the presence of macrophages, on activation of pattern recognition receptors such as Toll-like Receptor 9 (TLR9), on MyD88/TRIF signaling and on STAT1 expression in myeloid cells, and is characterized by accumulation of TER119^+^ cells of the red blood cell lineage in the spleen (19, 21–23). Of note, certain pathogens suppress extramedullary hematopoiesis as part of their immune evasion program (19), indicating an important role for this phenomenon in the innate immune defense. However, a mechanistic link between the response to infection and the massive expansion of erythroid TER119^+^ cells is elusive.

In this study, we investigated extramedullary hematopoiesis and the replenishment of the DC pool after infection. We identified inflammatory erythropoietin-dependent TER119^+^ cells in the spleen with the potential to differentiate into CD11c^high^ cells *in vitro* and *in vivo*. TER119 expression was transiently maintained on bona fide DC in the spleen and in the draining lymph nodes. Hence, our results describe a so far unknown pathway of inflammatory DC development in response to infection that is active during extramedullary hematopoiesis.

## Materials and Methods

### Mice

Female C57BL/6 mice were purchased from Elevate Janvier (Le Genest Saint Isle, France) at the age of 7 weeks and housed at the animal facility of the Max von Pettenkofer-Institute for at least 1 week before being used in experiments. TLR9^−/−^ mice (24) and ΔDC mice (25) were bred at the animal facilities of the II. Medizinische Klinik, Klinikum Rechts der Isar, Technical University Munich, and the Institute for Immunology, Ludwig-Maximilians-University Munich, respectively. ΔDC mice were generated by crossing CD11cCre mice (26) to mice that express the diphteria toxin α chain (DTA) under control of a loxP-flanked stop cassette from the ROSA26 locus (R-DTA mice) (27) leading to direct expression of DTA in DC and constitutive elimination of more than 95% of all cDCs and pDCs (25). The DC compartment remains depleted during inflammation (28). Experiments with knockout mice were performed with sex- and age-matched animals. Mice were housed at specified pathogen–free (SPF) health status in individually ventilated cages at 21–22°C and 39–50% humidity in groups of 5 animals. Animal experiments were approved by the responsible state government.

### Treatments

Experiments were performed with a dose of 20 nmol CpG-ODN 1826 (Tib Molbiol, Berlin, Germany) which reproducibly induced moderate extramedullary hematopoiesis, as in previous studies (23). The mice were anesthetized with isoflurane and CpG-ODN was injected subcutaneously (s.c.) into the right hind footpad in order to model local infection, as previously described (29). For *in vivo* blockade of EPO, the mice were injected intravenously (i.v.) with 250 μg monoclonal rat anti-mouse EPO antibody (clone 148438; cat#MAB959) or rat immunoglobulin G (IgG)2a isotype control (clone 54447; cat#MAB006) (R&D Systems) in phosphate-buffered saline (PBS) at day 2 and day 4, as described before (22). For CD115 blockade, mice were injected with 250 μg *in vivo* Ready™ anti-mouse CD115 antibody (anti-CSF-1R, clone AFS98; cat# 40-1152) and *in vivo* Ready™ Rat IgG2a Isotype Control (clone 2A3; cat# 40-4321) (Tonbo biosciences) i.v. at days 0, 2, and 4 post CpG-ODN treatment. For the adoptive transfer of TER119^+^CD11a^+^ cells, footpad injection was performed as described above in congenic wt and ΔDC animals. On day 6, TER119^+^CD11c^−^CD11a^+^ cells were harvested from the wt animals and transferred via tail vein injection to the ΔDC animals. Each animal received a transfer of 1.5 × 10^6^ cells.

### Infections

Pathogen infections were performed as follows: vaccinia virus Western Reserve, 10^5^ plaque-forming units (PFU) intraperitoneally (i.p.) (30); MCMV (bacterial artificial chromosome pSM3fr-derived Smith strain), 10^6^ PFU i.v. (19); MHV-68, 5 × 10^4^ PFU intranasally (i.n.) after ketamine/xylazine anesthesia (31); *L. monocytogenes* (strain ΔactA), 5 × 10^3^ colony-forming units (CFU) i.v. (19); and *P. aeruginosa* (strain PA01), 2 × 10^6^ CFU i.v.

### Cell Staining and Sorting

In order to obtain single cell suspensions, spleens and lymph nodes were cut into pieces and digested with 400 U/ml Collagenase D (Roche) and 100 μg / ml DNase I (Roche) in RPMI 1640 medium for 1 hour at 37°C. EDTA to a concentration of 0.01 M was added for 5 min to stop the enzymatic reactions. The digest was passed through a 70 μm cell strainer and cells were washed with PBS at 300 g for 7 min. In order to lyse mobile mature erythrocytes for the analysis of organ-resident TER119^+^ cells, we used ACK lysing buffer (155 mM ammonium chloride, 10 mM potassium bicarbonate, 0.1 mM EDTA, adjusted to pH = 7.2–7.4 using hydrochloric acid) in all of our experiments. For flow cytometry and cell sorting, cells were incubated with Mouse BD Fc Block™ (BD Pharmingen) in FACS buffer, washed, and stained with monoclonal antibodies specific to CD3e (clone 145-2C11) conjugated with PerCP-Cy5.5, CD11a (M17/4) PE and APC, CD11b (M1/70) FITC, CD11c (N418) APC, CD19 (eBio1/D3) APC, CD41 (MWReg30) FITC, CD71 (RI7 217.1.4) PE, CD115 (AFS98) PerCP-Cy5.5, CD117 (c-kit; 2B8) eFluor710, CD135 (A2F10) PE, CD172a (Sirp-α; P84) APC, Gr-1 (RB6-8C5) FITC, TER119 (TER-119) APC and PE, B220 (RA2-6B2) PE, Sca-1 (D7) PerCP-Cy5.5, MHC-II (M5/114.15.2) FITC, and NK 1.1 (PK136) PE for 20 min on ice (all antibodies were purchased from eBiosciences). After staining, cells were washed twice at 300 g for 7 min and resuspended in buffer containing propidium iodide (PI) for exclusion of dead cells. Cell acquisition was performed on a FACSCalibur™ or a FACSCanto II™ unit (Becton Dickinson) using CellQuest Pro™ or FACSDiva™ software, and data were analyzed using FlowJo (Tree Star Inc.). Cell sorting was performed on a FACSAria™ unit (Becton Dickinson).

### Cytokine Detection

For DC IL-12 production, splenocyte-derived DC (SDDC) and bone marrow-derived DC (BMDC) were plated in 6-well dishes at a density of 1 × 10^6^ cells / well and were stimulated for 12 h with CpG-ODN. Cell culture supernatants were collected and stored at −80 °C until analysis. For detection of IL-12p40 a commercially available kit (R&D; cat#MP400) was used according to the instructions of the manufacturer. For EPO detection, blood serum was isolated using Z-Gel 1.1 ml serum preparation tubes (Sarstedt, cat# 41.1378.005) according to the manufacturer's instructions. Serum samples were probed for EPO using a commercially available kit (R&D; cat#MEP00B) according to the instructions of the manufacturer.

### *In vitro* Differentiation Assays

DC were generated from spleens following the protocol for BMDC production with GM-CSF or Flt3-L (32). In brief, femora and tibiae were removed and cleaned of muscle tissue, disinfected for 3 min in 70% ethanol and then rinsed with PBS. The bones were cut with scissors at the epiphyses, the marrow was flushed with PBS using a sterile 27-gauge needle and passed through a 70 μm cell strainer. Erythrocytes were lysed in bone marrow and splenocyte single cell suspensions with ACK lysing buffer for 3 min at room temperature, the cells were washed once and plated at a density of 4 × 10^5^ cells / ml in 10 ml RPMI supported with 200 U/ml GM-CSF (PeproTech, cat#315-03) on 100 mm diameter Petri dishes. At day 3 of culture another 10 ml medium with 200 U/ml GM-CSF were added. After 6 days 10 ml of cell culture were removed, cells pelletized, re-suspended in 10 ml fresh medium containing 200 U/ml GM-CSF and retransferred to the remaining culture. For culture with Flt3-L, cells were plated in 10 ml RPMI with 20 ng / ml Flt3-L (PeproTech, cat#250-31L) at a density of 1.8 × 10^6^ cells / ml on 100 mm diameter Petri dishes and FLT-3L cultures were analyzed at day 10 without any further treatment. Colony formation assays were performed using MethoCult GF M3434 following the manufacturer's instructions (STEMCELL Technologies).

### Cytospins and Giemsa Staining

For cytospins, 8 × 10^4^ cells in PBS were spun onto microscope slides at 750 rpm for 2 min with slow acceleration using a Cytospin III (Shandon). Cells were fixed for 5 min in methanol before staining with a Giemsa-solution for 20 min. After washing with water microscope slides were air-dried and stored at 4°C.

### Magnetic Bead Enrichment

At day 10 after CpG-ODN footpad injection, single cell suspensions from draining popliteal lymph nodes were enriched for CD11c^+^ cells using mouse CD11c MicroBeads UltraPure according to the manufacturer's instructions (Miltenyi Biotec).

### Quantitative Reverse Transcription-PCR (qRT-PCR)

For analysis of *epor* and *gypa* expression TaqMan gene expression assays Mm 00833882_m1 and Mm 00494848_m1 were used, respectively.

### Statistics

Statistical analysis was performed using Prism5 (GraphPad Software).

## Results

### Sterile Inflammation Induces the Expansion of Erythropoietin-Dependent DC in the Spleen

**Figure 1:**
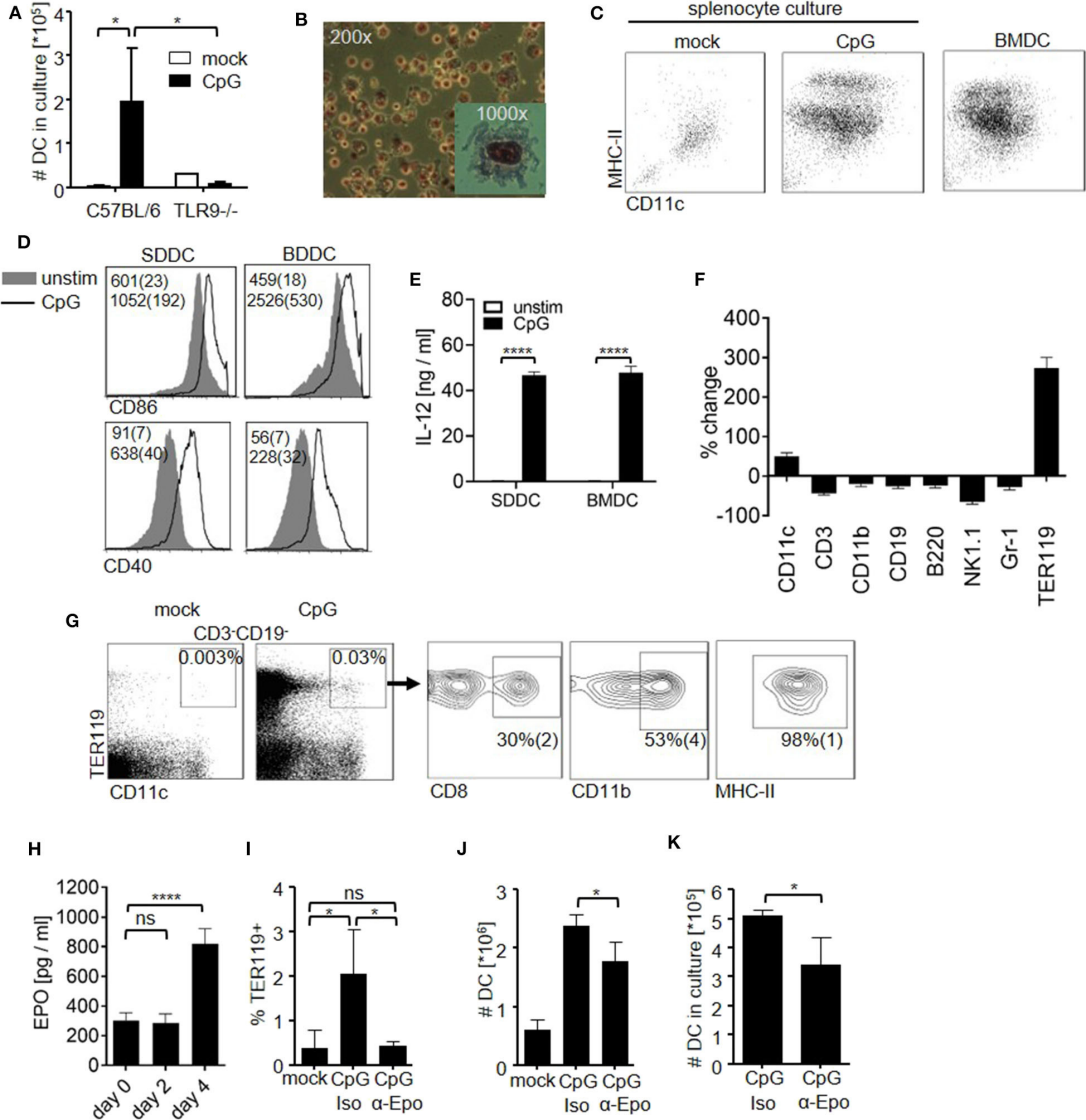
CpG-ODN stimulation induces an EPO-dependent increase in DC in the spleen. **(A–E)** C57BL/6 controls and TLR9-deficient mice (TLR9^−/−^) received a single dose of CpG-ODN. At day 6 post stimulation, harvested spleens were homogenized and splenocytes (4 × 10^5^ cells/ml) were cultured with GM-CSF for 10 days. **(A)** Graph shows number of DC obtained in culture. **(B)** Giemsa staining of splenocyte culture derived from CpG-ODN–treated C57BL/6 mice. **(C)** Surface phenotype of cells from splenocyte culture as determined by flow cytometry. DC generated from bone marrow (BMDC) were included as controls. Representative dot blots are shown. **(D,E)** DC derived from splenocyte culture (SDDC) and from bone marrow culture (BMDC) were stimulated with CpG-ODN. **(D)** Representative histograms show expression of CD86 and CD40 on DC. Numbers indicate MFI (median (SD)). **(E)** Bar graphs show IL-12p40 in cell culture supernatant. **(F,G)** C57BL/6 mice were stimulated with CpG-ODN and splenocytes were examined by flow cytometry at day 6 post treatment. **(F)** Splenocytes were stained for canonical lineage markers. Graph shows %-change of indicated populations compared to untreated controls. **(G)** Blots show surface phenotype of CD3^−^CD19^−^ splenocytes. Data of the animal representing the median of *n* = 5 animals are shown. **(H)** EPO levels in blood serum at the indicated time points after CpG-ODN treatment. **(I–K)** C57BL/6 mice were stimulated with CpG-ODN and received monoclonal anti-erythropoietin antibody (α-Epo) or isotype control (Iso). Graphs show **(I)** proportion of TER119^+^ cells in the splenic DC compartment, **(J)** total number of DC in the spleen at day 6 post stimulation and **(K)** number of DC after culture of splenocytes (5 × 10^5^ cells/ml) with GM-CSF for 10 days. **(A–K)** Representative data of at least 3 experiments with *n* = 3–5 animals/group are shown. **(A,E,H–K)** Bar graphs show mean (SD). One-way ANOVA with Bonferroni's post test **(A,I)** or Student's t test were performed **(E,H,J,K)**. Statistical significance is indicated by * = *p* < 0.05, **** = *p* < 0.0001, ns = *p* > 0.05.

Given the fact that many pathogens target DC (11–15), we hypothesized that the DC pool has to be replenished upon inflammation. In order to evaluate the spleen's DC generation potential in the course of inflammation, we stimulated mice with the synthetic TLR9 agonist CpG-oligodeoxynucleotide (CpG-ODN). Six days later, we harvested the spleens of stimulated and control mice and cultured the splenocytes for 10 days in the presence of granulocyte–monocyte colony-stimulating factor (GM-CSF). Consistent with an inflammation-induced stimulatory effect, cultures from CpG-ODN–treated animals yielded significantly more cells than cultures from control or TLR9-deficient animals (Figure 1A). The obtained cells (splenocyte-derived dendritic cells; SDDC) presented the distinct morphology of DC (Figure 1B) and could be separated into immature and mature populations according to the expression of major histocompatibility complex (MHC) class II molecules like DC generated from bone marrow culture (BMDC) (Figure 1C and Figures S1A,B). In addition, after overnight stimulation with CpG-ODN, SDDC up-regulated co-stimulatory molecule CD40 and CD86 expression and produced IL-12 amounts similar to BMDC (Figures 1D,E). Hence, we asked whether CpG-ODN-induced inflammation also resulted in expansion of the DC compartment *in vivo*. Strikingly, the proportion of CD11c^high^ splenocytes at day 6 following CpG-ODN stimulation increased by ~35% (Figure 1F and Figure S1C). All other lineages examined contracted proportionally upon CpG-ODN injection, except for TER119^+^ cells, which dominated the cellularity of the spleen after CpG-ODN injection. TER119 is expressed during late stages of red blood cell differentiation, i.e., from early proerythroblasts to mature erythrocytes (33). Nevertheless, we tested whether increased number of CD11c^+^ and TER119^+^ cells were linked and found a distinct TER119^+^CD11c^high^ population that appeared in CpG-ODN–stimulated spleens but not in the bone marrow (Figure 1G and Figures S1D,F). Additional analysis revealed that these TER119^+^CD11c^high^ splenocytes expressed MHC class II and either CD8 or CD11b similar to conventional DC in the spleen.

Erythropoietin (EPO) is known to expand the TER119^+^ cell compartment. Since TER119^+^ cell numbers increased upon sterile inflammation, we probed for EPO serum levels after CpG-ODN injection (Figure 1H). CpG-ODN administration significantly increased EPO levels at day 4 after stimulation, similar to what was observed after bacterial infection (22). In order to study the role of increased EPO levels during inflammation for the generation of TER119^+^CD11c^high^ cells, we stimulated mice with CpG-ODN and depleted EPO *in vivo* using a monoclonal antibody. EPO depletion abolished the increase in TER119^+^ cells and resulted in complete loss of TER119^+^CD11c^high^ cells (Figure 1I). Of note, depletion of EPO also resulted in a significant decrease in total DC numbers in the spleen upon CpG-ODN administration (Figure 1J). Although clearly additional EPO-independent DC generation mechanisms are in place after treatment with CpG-ODN, these data implicate EPO as a positive factor in DC development during inflammation. We confirmed this finding *in vitro* by culturing splenocytes from CpG-ODN–treated mice and found that *in vivo* EPO-depletion significantly reduced the number of DC generated from GM-CSF culture (Figure 1K). In summary, we found that sterile inflammation increased systemic EPO levels and enhanced the DC generation capacity in the spleen by EPO-dependent and -independent mechanisms.

### Inflammation Induces TER119^+^CD11a^+^ Cells That Can Differentiate Into Erythroid or Myeloid Cells

**Figure 2:**
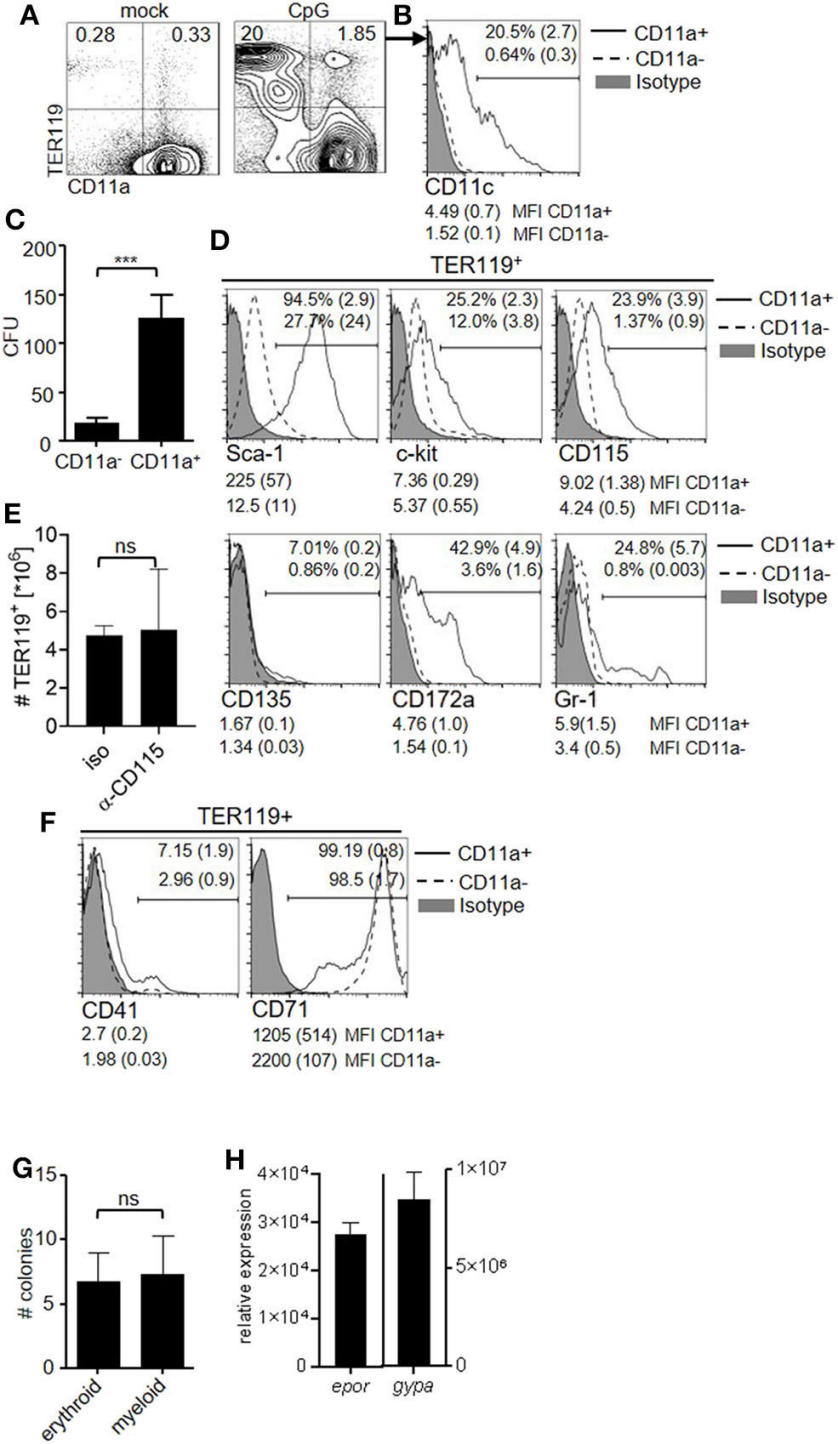
The TER119^+^CD11a^+^ cell pool harbors potential to differentiate into myeloid cells. **(A–H)** C57BL/6 mice were stimulated with CpG-ODN. **(A)** Blots show surface phenotype of splenocytes at day 6 post treatment. Data of the animal representing the median of *n* = 5 animals are shown. **(B)** Representative histogram showing surface expression of CD11c on TER119^+^CD11a^−^ and TER119^+^CD11a^+^ splenocytes at day 6 post CpG-ODN treatment. Proportion of CD11c^+^ cells (mean (SD)) and MFI (median (SD)) of CD11c expression on the indicated populations are shown. **(C)** Colony formation assay from TER119^+^CD11c^−^CD11a^−^ and TER119^+^CD11c^−^CD11a^+^ cells that were sorted from the spleens of CpG-ODN–stimulated mice. **(D)** Representative histograms showing surface phenotype of TER119^+^CD11a^−^ and TER119^+^CD11a^+^ splenocytes at day 6 post treatment for myeloid progenitor cell markers. Proportion of marker positive cells (mean (SD)) and MFI (median (SD)) of the respective markers on the indicated populations are shown. **(E)** CpG-ODN-treated mice were injected with anti-CD115 antibody or isotype control. Graph shows number of TER119^+^ cells at day 6 post CpG-ODN treatment. **(F)** Representative histograms showing surface phenotype of TER119^+^CD11a^−^ and TER119^+^CD11a^+^ splenocytes at day 6 post CpG-ODN treatment for erythroid cell markers. Proportion of CD41^+^ cells and CD71^+^ cells (mean (SD)) and MFI (median (SD)) of CD41 and CD71 expression on the indicated populations are shown. **(G)** Colony formation assay with TER119^+^CD11a^+^ cells cultured with EPO or GM-CSF. **(H)** Relative expression of *epor* and *gypa* transcripts in CD11c^+^ cells derived from TER119^+^CD11a^+^ cells after culture with GM-CSF for 10 days compared to BMDC. **(A–H)** Representative data of at least 3 experiments with *n* = 4–5 animals/group are shown. **(C,E,G,H)** Bar graphs show mean (SD). Student's t test was performed. Statistical significance is indicated by *** = *p* < 0.0001, ns = *p* > 0.05.

Because EPO contributed to the capacity of the spleen to generate DC and was required for the occurrence of TER119^+^CD11c^high^ cells during inflammation, we investigated whether TER119^+^ cells accumulating in the spleen after TLR9-ligation could differentiate into DC *in vitro*. We observed that CD11a, the α_L_ subunit of the integrin LFA-1, discriminated two populations, i.e., TER119^+^CD11a^−^ and TER119^+^CD11a^+^, in the inflammation-induced TER119^+^ compartment (Figure 2A), as was previously shown (34). A subpopulation among TER119^+^CD11a^+^ cells already expressed low to intermediate levels of CD11c (Figure 2B). To exclude cells that were already positive for CD11c from our further analysis, we sorted TER119^+^CD11c^−^CD11a^−^ and TER119^+^CD11c^−^CD11a^+^ cells from the spleen of CpG-ODN–stimulated animals and cultured them with GM-CSF for 10 days (Figure S1E). The TER119^+^CD11c^−^CD11a^+^ cells had 5-fold greater capacity to generate CD11c^+^ myeloid colonies than the TER119^+^CD11c^−^CD11a^−^ cells (Figure 2C). As TER119^+^CD11c^−^CD11a^+^ cells showed potential to differentiate into myeloid cells, we analyzed these cells for surface markers that are expressed by myeloid progenitor cells (Figure 2D). We found expression of Sca-1, c-kit, CD172a (Sirp-α) and CD115 (MCSFR) on TER119^+^CD11a^+^ cells isolated from the spleen, but not on TER119^+^CD11a^−^ cells. CD135 (FLT-3) and Gr-1 expression was only detectable on a small proportion of TER119^+^CD11a^+^ cells. CD115 differentially controls the development of myeloid cell populations (35). In order to test whether CD115 blockade affects the expansion of TER119^+^ cells, we administered anti-CD115 antibody. As expected, CD115 blockade reduced the numbers of splenic macrophages but not of DC (Figure S2) (36). The number of TER119^+^ cells after CpG-ODN treatment was not affected by anti-CD115 administration (Figure 2E), excluding a major role for CD115 in the expansion of the TER119^+^ cell compartment.

Because TER119^+^CD11a^+^ cells expressed with TER119 a red blood cell marker, we probed for further markers of early and late stages of erythroid development. Only a minor fraction of TER119^+^CD11a^+^ cells expressed CD41 (platelet glycoprotein IIb [alpha(IIb)]), a marker of early erythropoiesis, while late stage erythroid marker transferrin receptor CD71 was highly expressed (Figure 2F). Due to the mixed surface phenotype of erythroid and myeloid progenitor cells displayed by TER119^+^CD11a^+^ splenocytes, we tested whether these cells present the ability to differentiate into both myeloid and erythroid lineages. We performed colony formation assays and cultured TER119^+^CD11a^+^ splenocytes with EPO or GM-CSF. The number of colonies generated was comparable under both conditions (Figure 2G). We asked whether the obtained CD11c^+^ myeloid cells in culture retained erythroid marker gene expression and probed for expression of EPO receptor (*epor*) and glycophorin A (*gypa*) transcripts by quantitative reverse transcription–PCR (qRT-PCR). CD11c^+^ myeloid cells derived from TER119^+^CD11a^+^ splenocytes had 30,000- and 8,000,000-fold higher expression of *epor* and *gypa*, respectively, compared to DC derived from bone marrow (Figure 2H).

In summary, our data suggest that inflammation induces the expansion of TER119^+^CD11a^+^ cells in the spleen that have the potential to differentiate into myeloid cells.

### TER119^+^CD11c^high^ Cells Contribute to the DC Compartment Upon Local Inflammation

**Figure 3:**
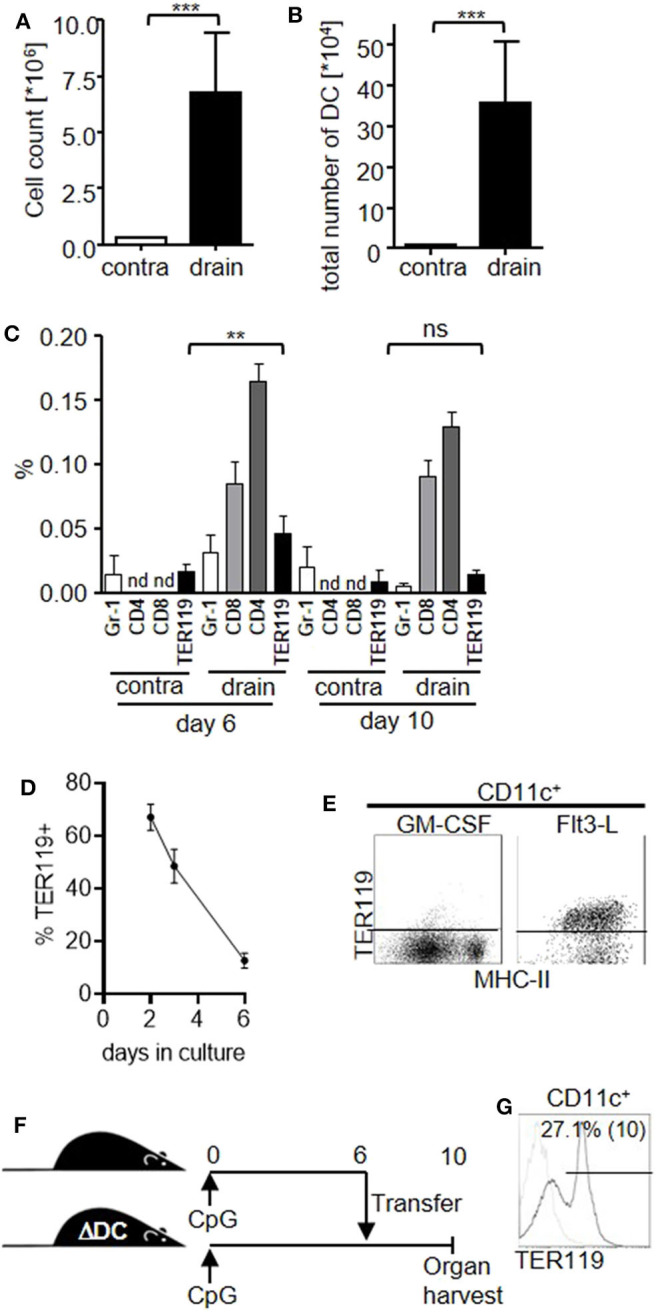
TER119^+^CD11c^high^ cells contribute to the DC compartment in the draining lymph node upon local inflammation. **(A–C)** Mice were injected with a single dose of CpG-ODN into one footpad. Graphs show **(A)** cell count of the contralateral (contra) and draining (drain) lymph nodes at day 10 post stimulation, **(B)** total number of DC in lymph nodes, **(C)** proportion of B220^−^CD11c^+^CD11b^high^ Gr-1^+^ (Gr-1), CD4^+^, CD8^+^ and TER119^+^CD11c^+^ DC subsets in the lymph nodes at day 6 and day 10 post stimulation. nd = not examined. Representative data of at least 3 experiments with *n* = 5 animals/group are shown. **(D,E)** TER119^+^CD11a^+^ splenocytes from CpG-ODN treated mice were cultured with GM-CSF or FLT3-L. **(D)** Graph shows proportion of TER119^+^ cells in culture with GM-CSF at the indicated time points. **(E)** Representative blots showing TER119 and MHC-II expression on CD11c^+^ cells after culture with Flt3-L. **(F)** Experimental setup for the adoptive transfer of TER119^+^CD11a^+^ splenocytes from congenic wt mice into CpG-ODN–stimulated mice that are deficient for DC (ΔDC). **(G)** Donor-derived cells from the draining lymph node were gated for CD11c. Representative histogram shows TER119 expression and frequency of TER119^+^ cells within the CD11c gate (mean (SD)). *n* = 3 animals. **(A–D)** Bar graphs show mean (SD). **(A–C)** Student's t test was performed. Statistical significance is indicated by ** = *p* < 0.01, *** = *p* < 0.0001, ns = *p* > 0.05.

Having shown the capacity of TER119^+^CD11a^+^ splenocytes to differentiate into CD11c^+^ myeloid cells, we wanted to know whether this differentiation pathway would also be active in the local inflammatory environment. We injected CpG-ODN subcutaneously into mouse footpads and observed substantially increased cellularity in the draining lymph nodes, peaking at day 10 post injection (Figure 3A), associated with an increased number of DC (Figure 3B), as was previously shown (29). We analyzed the DC compartment in the draining lymph node following CpG-ODN stimulation and found that TER119^+^CD11c^high^ cells were proportionally increased at day 6 post treatment (Figure 3C). Hence, inflammation-induced TER119^+^CD11c^high^ cells are detectable both in the spleen and in the peripheral lymph nodes *in vivo*.

Interestingly, TER119^+^CD11c^high^ cells represented only a minor proportion of the whole DC population in the draining lymph nodes compared to CD8^+^ or CD4^+^ DC subsets. Furthermore, TER119^+^CD11c^high^ cells peaked in the lymph nodes at day 6 and decreased until day 10, when mature DC numbers peaked. Also, the expression level of TER119 on lymph node DC was low compared to TER119^+^CD11a^+^ splenocytes or TER119^+^CD11c^high^ cells in the spleen (Figure S3). Therefore, we hypothesized that TER119^+^CD11c^high^ cells further differentiate and lose their erythroid marker. In fact, we observed that TER119^+^CD11a^+^ splenocytes gradually lost TER119 expression during differentiation with GM-CSF *in vitro* (Figure 3D), while cultures supported by Flt3-L maintained TER119 expression during conversion to immature CD11c^+^ cells (Figure 3E).

However, we were unable to detect TER119^+^CD11a^+^ cells in lymph nodes following CpG-ODN treatment. To explore whether TER119^+^CD11c^high^ cells in the draining lymph nodes could be derived from TER119^+^CD11a^+^ found in the spleen, we transferred TER119^+^CD11a^+^ splenocytes from wild-type (wt) donor mice to recipient mice that are constitutively depleted of DC due to diphtheria toxin expression in CD11c^+^ cells (ΔDC mice) and therefore present an empty niche (Figure 3F) (25). We injected CpG-ODN into the footpads of both the congenic wt donor and ΔDC recipient mice to create the local inflammatory environment. At day 6 post stimulation, we transferred TER119^+^CD11a^+^ splenocytes from the wt animals to the ΔDC recipients, and at day 10 we analyzed the draining lymph node. We detected donor-derived CD11c^+^ cells only in ΔDC mice that had received TER119^+^CD11a^+^ cells. A proportion of these cells retained TER119 expression (Figure 3G), suggesting that TER119^+^CD11a^+^ splenocytes could differentiate into TER119^+^CD11c^+^ and TER119^−^CD11c^high^ cells in the draining lymph nodes *in vivo*.

### Infection-Induced Inflammation Generates TER119^+^CD11c^high^ Cells

**Figure 4:**
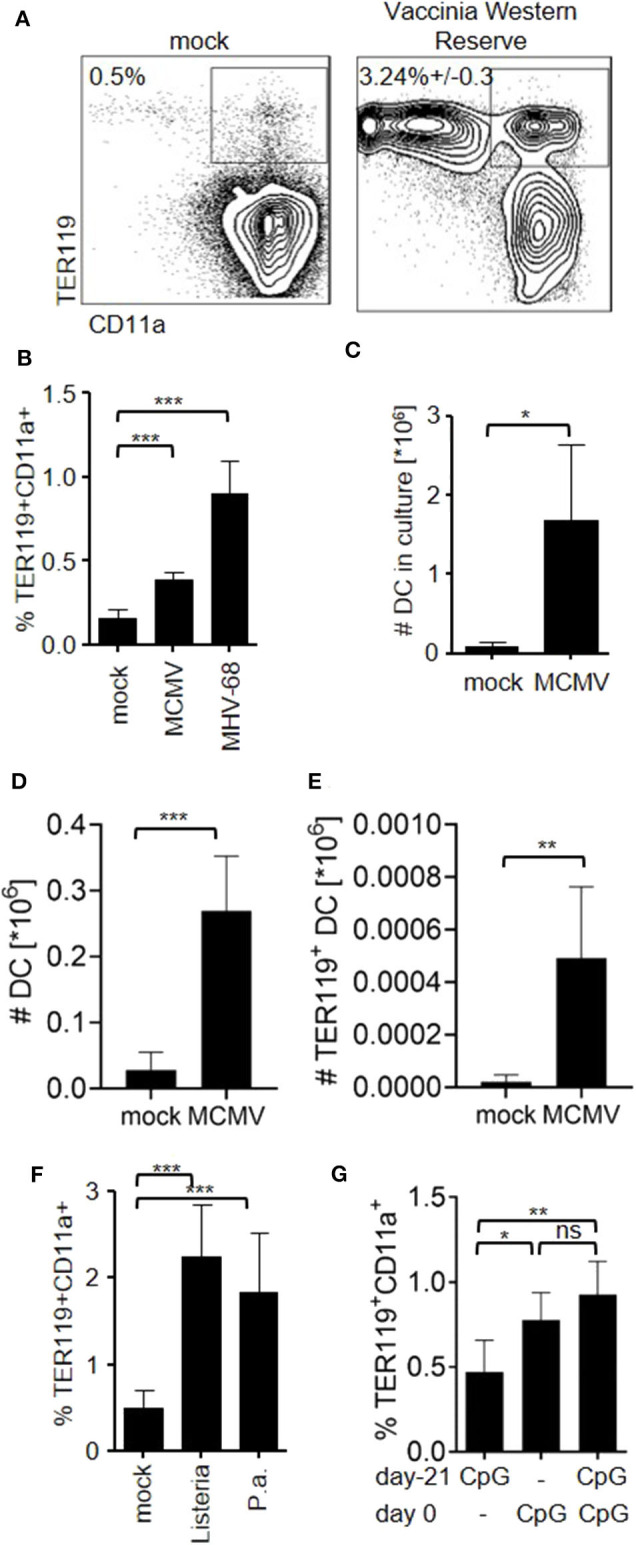
Viral and bacterial infections induce the expansion of TER119^+^ inflammatory DC. **(A)** Mice were infected with vaccinia virus Western Reserve. Blots show TER119 and CD11a expression on splenocytes at day 6 post-infection. Data of the animal representing the median of *n* = 5 animals are shown. **(B–E)** Mice were infected with mouse cytomegalovirus (MCMV) or MHV-68. Graphs show **(B)** proportion of TER119^+^CD11a^+^ splenocytes at day 6 (MCMV) and day 17 (MHV-68) post-infection, **(C)** number of DC in splenocyte culture from MCMV-infected mice after 10 days with GM-CSF, **(D)** number of DC in spleens, and **(E)** number of TER119^+^CD11c^+^ cells in the spleen after infection with MCMV. **(F)** Mice were infected with *Listeria monocytogenes* (Listeria) and *Pseudomonas aeruginosa* (P.a.). Graphs show proportion of TER119^+^CD11a^+^ cells in the spleen. **(G)** Mice were stimulated with CpG-ODN. Three weeks later, mice were left untreated or received a second dose of CpG-ODN, and were compared to naïve mice receiving CpG-ODN. Bar graphs show proportion of TER119^+^CD11a^+^ cells at day 6 after second stimulation. **(B–G)** Representative data of at least 3 experiments with *n* = 5 animals/group are shown. Bar graphs show mean (SD). Student's t test was performed. Statistical significance is indicated by * = *p* < 0.05, ** = *p* < 0.01, *** = *p* < 0.0001.

Finally, we examined whether expansion of TER119^+^CD11a^+^ cells and TER119^+^CD11c^high^ cells was a result of isolated TLR9-ligation with CpG-ODN or would also occur during infection-induced inflammation. After infection with a poxvirus (vaccinia virus Western Reserve), a β-herpesvirus (murine cytomegalovirus, MCMV) and a murine γ-herpesvirus (murine gammaherpesvirus 68, MHV-68) TER119^+^CD11a^+^ cells became readily detectable in the spleen (Figures 4A,B). Accordingly, splenocyte cultures from MCMV-infected mice yielded more DC *in vitro* (Figure 4C), and more DC including TER119^+^CD11c^high^ cells could be detected *in vivo* (Figures 4D,E). Bacterial pathogens such as intracellular *Listeria monocytogenes* and extracellular *Pseudomonas aeruginosa* also expanded TER119^+^CD11a^+^ cells (Figure 4F). Finally, we tested whether induction of extramedullary hematopoiesis only occurs once in naïve individuals, or re-occurs after repeated TLR-ligation. We stimulated mice with CpG-ODN and repeated the procedure after three weeks (Figure 4G). The proportion of TER119^+^CD11a^+^ splenocytes in mice that had received CpG-ODN twice and in mice that were stimulated once was comparable, indicating that TLR ligation can trigger extramedullary hematopoiesis recurrently. In summary, we have found that inflammation induced EPO-dependent TER119^+^CD11a^+^ cells that could differentiate into TER119^+^ and TER119^−^ DC contributing to the replenishment of the DC pool in the spleen and draining lymph nodes after infection.

## Discussion

Dendritic cells are critical for the initiation of adaptive immune responses. Consequently, many bacteria and viruses specifically target DC causing functional paralysis or deletion of DC in order to evade eradication (11, 13–15, 37). As a result, DC populations must be replenished following pathogen infection to ensure operational readiness of the adaptive immune system. In fact, we found an increased potential of spleen homogenates to generate DC *in vitro* and increased splenic DC numbers *in vivo* after stimulation of mice with synthetic TLR9 ligand CpG-ODN. Most likely several myeloid progenitors contribute to the expansion of the DC compartment upon inflammation. We observed an increase in TER119^+^ cells indicating the activation of extramedullary hematopoiesis, as was described after stimulation with TLR7 and TLR9 ligands as well as after infection with bacteria (*Salmonella spp*., *Ehrlichia spp*.) and virus (mouse cytomegalovirus) (19, 21–23, 38–40). Subsequently, we found that TER119 was also detectable on the surface of a small proportion of CD11c^high^ cells. These TER119^+^CD11c^+^ cells expressed MHC class II and CD8 or CD11b, therefore exhibiting the distinct surface markers of conventional DC. Though inflammation increased the numbers of TER119^+^CD11c^+^ cells, a low level of TER119^+^ expression on myeloid cells can also be observed in the steady-state (41).

Fulminant inflammation can result in a syndrome called hemophagocytosis, in which phagocytes engulf other hematopoietic cells. Thus, phagocytosis of erythrocytes or of TER119^+^ progenitor cells can result in a positive signal for TER119 after intracellular staining of DC (42, 43). We used surface staining to detect TER119 on the cell membrane of CD11c^high^ splenocytes upon inflammation. Nevertheless, it is conceivable that DC acquire TER119 from other cells through extravesicular particles, similar to the acquisition of MHC complexes by so-called MHC cross-dressing. Though these phenomena might contribute to phenotypically identical TER119^+^CD11c^+^ cells, Dash et al. (34) and we identified an inflammatory TER119^+^CD11a^+^ population in the spleen, that had the potential to give rise to erythroid or CD11c^+^ myeloid colonies depending on the cytokine profile that they were exposed to in our experiments. TER119^+^CD11a^+^ cells differentiated into functional DC *in vitro* if cultured with GM-CSF or FLT3-L, and into CD11c^high^ cells *in vivo* after transfer into DC-deficient mice, downregulating TER119 expression during this process, which suggests that TER119 expression on DC is a remnant of earlier TER119^+^ differentiation stages. Since co-expression of TER119 and CD11c might only be a short transient state, it may well be that the small number of TER119^+^CD11c^+^ cells underestimates the contribution of inflammation-induced TER119^+^CD11a^+^ cells to the DC compartment. Future studies using genetic tools will trace TER119 expression in CD11c^+^ cells. At this point it remains unclear whether individual TER119^+^CD11a^+^ cells have the plasticity to differentiate into both the erythroid or myeloid lineage, or if cells within this population are already committed for one of the lineages. From our data it appears that only a minor fraction of TER119^+^CD11a^+^ cells differentiated into DC, because not all seeded cells converted into DC *in vitro*, and peak TER119^+^CD11a^+^ cell numbers greatly exceed splenic DC numbers *in vivo*. Further investigations using single cell technologies will have to address the plasticity of TER119^+^CD11a^+^ cells.

Inflammation-induced extramedullary erythropoiesis depends on EPO. We found increased systemic EPO levels at day 4 after TLR9 ligation. EPO was absolutely required for the expansion of TER119^+^CD11a^+^ cells and TER119^+^CD11c^high^ cells in the spleen. It is conceivable that elevated EPO levels during inflammation induced TER119 expression on conventional CD8^+^ and CD11b^+^ DC subsets. However, TER119^+^ DC were mostly detectable in the draining lymph node after localized inflammation, while EPO levels increase systemically. Also, while EPO injection or overexpression of human EPO in transgenic mice expands the splenic DC pool (44), our data show that EPO depletion in CpG-ODN–treated mice decreased the total number of DC in the spleen significantly and reduced the number of DC obtained from splenocyte culture *in vitro*. In summary, these data suggest that EPO-dependent and -independent mechanisms contribute to DC development upon inflammation.

Finally, we were able to demonstrate that TER119^+^CD11c^high^ cells are induced not only during sterile inflammation but also after infection with bacterial and viral pathogens, for example after infection with cytomegalovirus, a pathogen known to induce extramedullary hematopoiesis and to impair DC function (13, 19, 45, 46). These findings led us to the conclusion that the expansion of TER119^+^CD11a^+^ cells is a general inflammatory mechanism triggered by viral or bacterial pathogens in order to maintain the DC compartment after infection. As part of the innate immune response to inflammation, it is conceivable that the induction of extramedullary hematopoiesis and TER119^+^CD11a^+^ cells occurs recurrently after infection. In fact, we have found that repeated CpG-ODN injection does not affect the expansion of the TER119^+^CD11a^+^ cell pool, suggesting that TLR ligation might trigger replenishment of the DC pool by default. CpG-ODN and mouse cytomegalovirus are recognized by TLR9 (47), and up to date only TLR7 and TLR9 have been shown to induce extramedullary hematopoiesis and TER119^+^ splenocytes (23, 39, 40). Nevertheless, pathogens produce numerous ligands for pattern recognition receptors. Interestingly, it was shown that IL-12 is sufficient for the induction of extramedullary hematopoiesis (48). Hence, IL-12 induction by pattern recognition receptor signaling might be the trigger for the induction of extramedullary hematopoiesis and the replenishment of the DC compartment.

It is important to note that TER119 is commonly used as a lineage marker to exclude erythroid cells in cell sorting experiments. This technicality and the fact that TER119^+^CD11c^high^ cells seem to be a transient differentiation state after an inflammatory stimulus probably explains why TER119^+^ DC have so far escaped attention. Taken together, our data indicate that EPO-dependent inflammatory myeloid cells in spleen and lymph nodes that are transiently marked by TER119 expression contribute to the replenishment of the DC pool in response to an infection.

## Data Availability Statement

The raw data supporting the conclusions of this article will be made available by the authors, without undue reservation.

## Ethics Statement

The animal study was reviewed and approved by Regierung von Oberbayern, licensing numbers: 70/08, 50/09, 97/12.

## Author Contributions

HE, AH, and AS performed experiments and analyzed the data. GG and HA provided materials, performed experiments, and analyzed the data. DV provided mice and intellectual expertise. MM provided intellectual expertise. ZR and LD provided intellectual expertise and wrote the paper. UK and TS provided intellectual expertise and funded the study. WR and SJ conceived the study, designed, performed and analyzed experiments, wrote the manuscript, and funded the study. All authors discussed the data and edited the manuscript.

## Conflict of Interest

The authors declare that the research was conducted in the absence of any commercial or financial relationships that could be construed as a potential conflict of interest.

## Supplementary Material

Figure S1 Splenocyte culture and lineage characterization in spleen after CpG-ODN stimulation. **(A,B)** C57BL/6 received a single dose of CpG-ODN. At day 6 post stimulation, harvested spleens were homogenized and splenocytes (4 × 10^5^ cells/ml) were cultured with GM-CSF. As control, bone marrow cells from untreated animals were cultured with GM-CSF. After 10 days in culture, splenocyte-derived DC (SDDC), and bone marrow-derived DC were analyzed (*n* = 3 animals/group). Bar graphs show **(A)** frequencies of MHC-II^high^ cells among CD11c^+^ cells, and **(B)** mean fluorescence intensity (MFI) of MHC-II^int^ and MHC-II^high^ populations (mean (SD)). **(C–E)** C57BL/6 mice were stimulated with CpG-ODN (CpG; *n* = 5 animals) and analyzed at day 6 post treatment or were left untreated (mock; *n* = 3 animals). Dot blots show gating strategy for splenocytes **(C)** stained with the indicated lineage markers, **(D)** upstream of TER119^+^CD11c^high^ cells, and **(E)** for sorting of TER119^+^CD11a^−^CD11c^−^ and TER119^+^CD11a^+^CD11c^−^ cell populations. Data of the animal representing the group median are shown. Numbers indicate mean proportion of gated cells (SD). **(F)** C57BL/6 mice were stimulated with CpG-ODN and bone marrow cells were examined by flow cytometry at day 6 post treatment. Dot blots show surface phenotype of CD3^−^CD19^−^ bone marrow cells. Data of the animal representing the median of *n* = 5 animals are shown.

Figure S2 Differential effect of CD115 blockade on myeloid cell populations. CpG-ODN-treated mice were injected with anti-CD115 antibody or isotype control. Graphs show numbers of macrophages and DC in spleen at day 6 post CpG-ODN treatment. *n* = 4 animals/group (mean (SD)). Student's t test was performed. Statistical significance is indicated by ^***^ = *p* < 0.0001, ns = *p* > 0.05.

Figure S3 Expression of TER119 on CD11c^+^ cells in the draining lymph node. Mice were injected with a single dose of CpG-ODN into one footpad. At day 10 post stimulation, the draining popliteal lymph nodes were harvested. Single cell suspensions from 5 mice were pooled and enriched for CD11c^+^ cells using magnetic beads. Dot blots show staining with antibodies against CD11c and with TER119 or isotype control antibody.
