# Supplementary figures and images for: The Innate Immune Response to Infection Induces Erythropoietin-Dependent Replenishment of the Dendritic Cell Compartment
Source: Front Immunol. 2020 Jul 31;11:1627. doi: 10.3389/fimmu.2020.01627 (PMC7411349; doi:10.3389/fimmu.2020.01627)

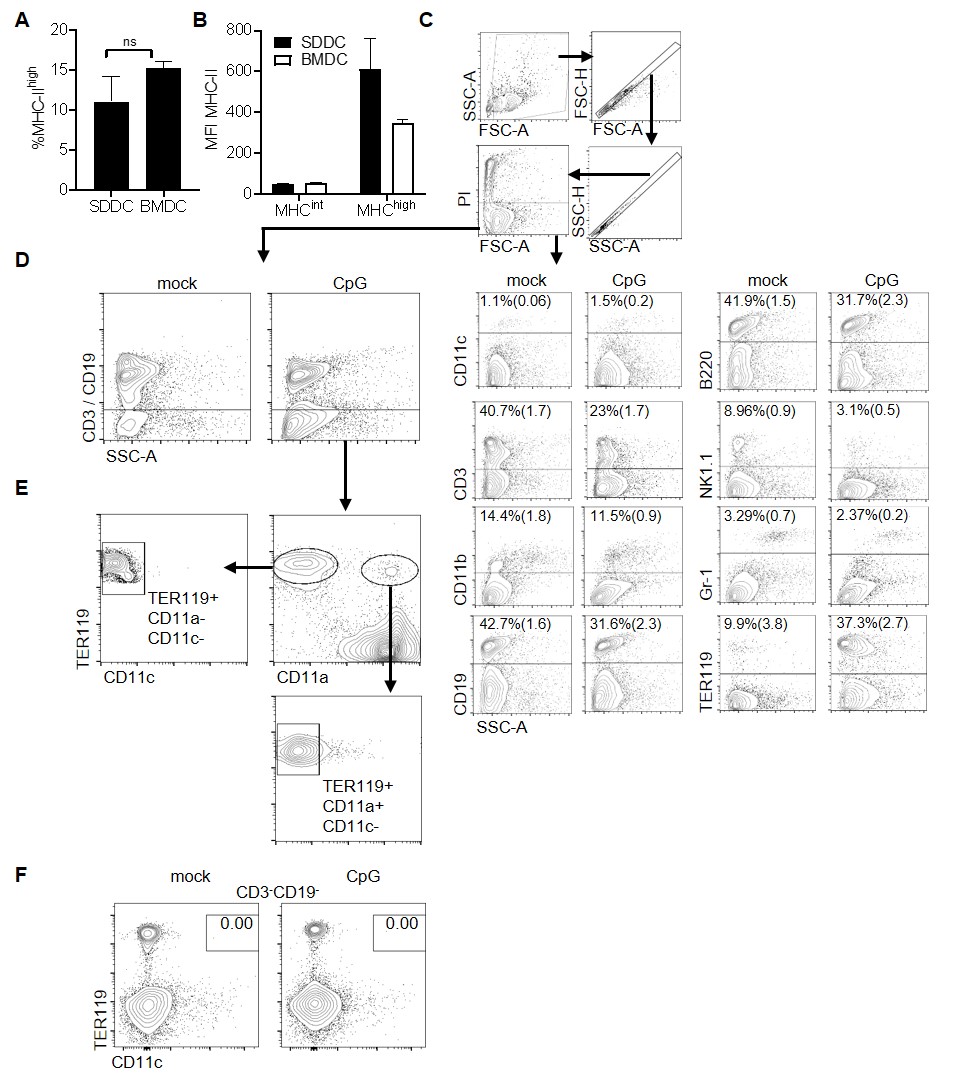

Supplement: Figure S1 — Splenocyte culture and lineage characterization in spleen after CpG-ODN stimulation. (A,B) C57BL/6 received a single dose of CpG-ODN. At day 6 post stimulation, harvested spleens were homogenized and splenocytes (4 × 10^5^ cells/ml) were cultured with GM-CSF. As control, bone marrow cells from untreated animals were cultured with GM-CSF. After 10 days in culture, splenocyte-derived DC (SDDC), and bone marrow-derived DC were analyzed (n = 3 animals/group). Bar graphs show (A) frequencies of MHC-IIhigh cells among CD11c+ cells, and (B) mean fluorescence intensity (MFI) of MHC-IIint and MHC-IIhigh populations (mean (SD)). (C–E) C57BL/6 mice were stimulated with CpG-ODN (CpG; n = 5 animals) and analyzed at day 6 post treatment or were left untreated (mock; n = 3 animals). Dot blots show gating strategy for splenocytes (C) stained with the indicated lineage markers, (D) upstream of TER119+CD11chigh cells, and (E) for sorting of TER119+CD11a−CD11c− and TER119+CD11a+CD11c− cell populations. Data of the animal representing the group median are shown. Numbers indicate mean proportion of gated cells (SD). (F) C57BL/6 mice were stimulated with CpG-ODN and bone marrow cells were examined by flow cytometry at day 6 post treatment. Dot blots show surface phenotype of CD3−CD19− bone marrow cells. Data of the animal representing the median of n = 5 animals are shown. [file Image_1.jpg]

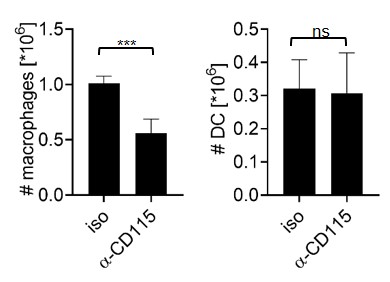

Supplement: Figure S2 — Differential effect of CD115 blockade on myeloid cell populations. CpG-ODN-treated mice were injected with anti-CD115 antibody or isotype control. Graphs show numbers of macrophages and DC in spleen at day 6 post CpG-ODN treatment. n = 4 animals/group (mean (SD)). Student's t test was performed. Statistical significance is indicated by *** = p < 0.0001, ns = p > 0.05. [file Image_2.JPEG]

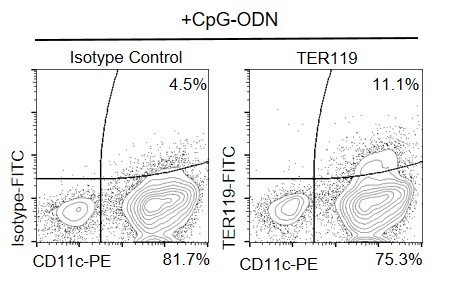

Supplement: Figure S3 — Expression of TER119 on CD11c+ cells in the draining lymph node. Mice were injected with a single dose of CpG-ODN into one footpad. At day 10 post stimulation, the draining popliteal lymph nodes were harvested. Single cell suspensions from 5 mice were pooled and enriched for CD11c+ cells using magnetic beads. Dot blots show staining with antibodies against CD11c and with TER119 or isotype control antibody. [file Image_3.JPEG]
